# Constrained Unscented Particle Filter for SINS/GNSS/ADS Integrated Airship Navigation in the Presence of Wind Field Disturbance

**DOI:** 10.3390/s19030471

**Published:** 2019-01-24

**Authors:** Zhaohui Gao, Dejun Mu, Yongmin Zhong, Chengfan Gu

**Affiliations:** 1 School of Automatics, Northwestern Polytechnical University, Xi’an 710072, China; mudejun@nwpu.edu.cn; 2 School of Engineering, RMIT University, Bundoora, Melbourne 3083, Australia; yongmin.zhong@rmit.edu.au; 3 Department of Mechanical Engineering, University of Melbourne, Parkville, Melbourne 3010, Australia; chengfan.gu@gmail.com

**Keywords:** airship navigation, SINS/GNSS/ADS integration, wind field disturbance, constrained unscented particle filter

## Abstract

Due to the disturbance of wind field, it is difficult to achieve precise airship positioning and navigation in the stratosphere. This paper presents a new constrained unscented particle filter (UPF) for SINS/GNSS/ADS (inertial navigation system/global navigation satellite system/atmosphere data system) integrated airship navigation. This approach constructs a wind speed model to describe the relationship between airship velocity and wind speed using the information output from ADS, and further establishes a mathematical model for SINS/GNSS/ADS integrated navigation. Based on these models, it also develops a constrained UPF to obtain system state estimation for SINS/GNSS/ADS integration. The proposed constrained UPF uses the wind speed model to constrain the UPF filtering process to effectively resist the influence of wind field on the navigation solution. Simulations and comparison analysis demonstrate that the proposed approach can achieve optimal state estimation for SINS/GNSS/ADS integrated airship navigation in the presence of wind field disturbance.

## 1. Introduction

The stratosphere, which is located at the bottom of the near space with the altitude between 10 km to 50 km, is a new region of human activities in space [1,2]. As a typical aircraft flying in the stratosphere, an airship has the advantages of high flight altitude, large coverage, and low-cost [2,3]. However, due to the disturbance of wind field in the stratosphere, airship positioning and navigation remain a challenging research problem.

The airship relies on an integrated navigation system for positioning and navigation. Inertial navigation system (INS)/global navigation satellite system (GNSS) integration is the most widely used integrated navigation system [4,5]. However, due to the near-far and ionosphere effects, satellite signals may be shielded or submerged. The signal reception rate declines to 60% or even lower in environments with valleys, underground areas, or business districts [5,6]. When satellite signals cannot be received normally, the error of INS will be accumulated over time, deteriorating the navigation accuracy [7,8,9].

The atmosphere data system (ADS) utilizes pressure, temperature, and geographic information (such as position calibration data, airspeed, airflow data, and time) provided by the atmospheric sensor to calculate the velocity and altitude of an aerial vehicle [10,11]. ADS does not rely on external conditions to obtain the navigation information. It has high reliability and its performance is not affected by height, topography, and other factors [11,12]. Given its advantages, ADS is an ideal auxiliary for INS/GNSS navigation to compensate the error of INS, especially when GNSS signal is poor, rendering INS/GNSS/ADS integration as a promising scheme to improve the navigation accuracy and reliability [13]. However, when using INS/GNSS/ADS integration for airship navigation, the navigation performance is significantly disturbed by wind field due to the long-span flexible balloon structure and low speed of the airship.

The performance of INS/GNSS/ADS integration is dominated by the filtering algorithm used [14,15,16]. The particle filter (PF) is an optimal recursive Bayesian filtering method based on Monte Carlo simulation by producing a sample of independent random variables according to a conditional probability distribution [17,18]. It is easy to implement, suitable for high-dimensional problems, and capable of handling nonlinear and non-Gaussian models [18,19]. However, the accuracy of PF relies on importance sampling. PF also requires the design of a proposal distribution to accurately approximate the posterior distribution, while in practice it is difficult to find such a proposal distribution [20,21].

The unscented Kalman filter (UKF) is able to generate a proposal distribution with larger high-order moment and the mean that is close to the true mean of the target distribution [22,23]. The combination of UKF into PF results in the so-called unscented particle filter (UPF), which is widely used in many fields including aircraft navigation, underwater navigation, GPS precise point positioning, nonlinear system identification, and audio source separation [24,25,26,27,28]. However, UPF is incapable of handling the disturbance of wind field for INS/GNSS/ADS integrated airship navigation. The robust adaptive UPF (RAUPF) is a method for system state estimation in the presence of abnormal observations and kinematic model noise [29,30]. This method adaptively determines the equivalent weight function according to robust estimation and adaptively adjusts the adaptive factor constructed from predicted residuals to inhibit the disturbances of abnormal observation and kinematic model noise. However, the wind field in the stratosphere has a unique form of disturbance, which is completely different from kinematic model noise and observation noise, requiring a special way to handle. Further, it cannot guarantee the covariance matrices in the filtering process are positive-definite, leading to the stability issue.

This paper presents a new constrained UPF for SINS/GNSS/ADS integrated airship navigation under the disturbance of wind field. A model of wind speed is established using ADS output information to describe the relationship between airship velocity and wind speed. Further, a mathematical model is also established for SINS/GNSS/ADS integrated airship navigation. Based on these models, a constrained UPF is developed to fuse SINS and GNSS measurements to generate the optimal state estimation for SINS/GNSS/ADS integration. This constrained UPF applies the wind speed model as a constraint to the UPF filtering process to effectively inhibit the influence of wind filed on the navigation solution. Simulations and comparison analysis have been conducted to comprehensively evaluate the performance of the proposed constrained UPF for SINS/GNSS/ADS integrated airship navigation in the presence of wind disturbance.

## 2. Mathematical Model of SINS/GNSS/ADS Integrated Navigation

### 2.1. Wind Speed Model

In the E–N–U (East-North-Up) geography coordinate system, the wind speed model can be expressed as

(1) $$v_{W}=v_{Wc}+v_{Wr}$$

where, $v_{W}$ is the wind speed, $v_{Wc}$ is a random constant wind, and $v_{Wr}$ is a random wind expressed as the first-order Markov process, and

**Figure 1:**
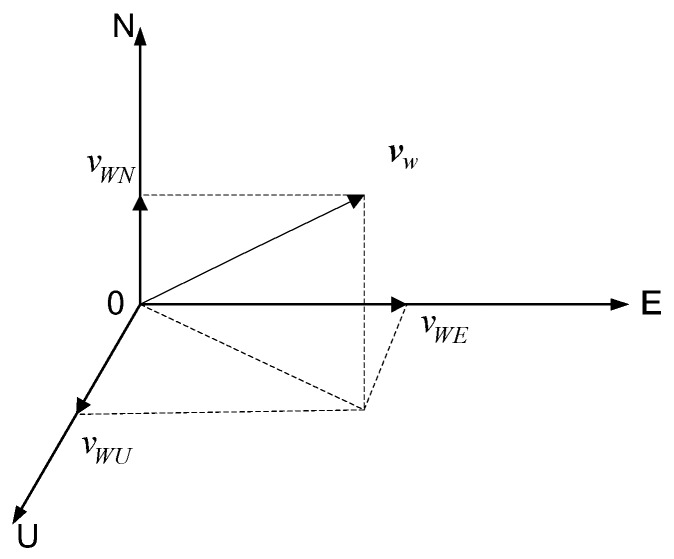
The components of the wind speed in the coordinate system E–N–U.

(2) vWE=vWcE+vWrEvWN=vWcN+vWrNvWU=vWcU+vWrUv˙Wci=0v˙Wri=−1τWrivWri+ωi

where $v_{Wi}(i=E,N,U)$ stands for the projection of wind speed $v_{W}$ in the East, North, and Up directions, as shown in Figure 1; the symbols $v_{Wci}$ and $v_{Wri}$ have the similar meanings as $v_{Wi}$; and $\omega_{i}(i=E,N,U)$ is the white noise in the corresponding direction.

**Figure 2:**
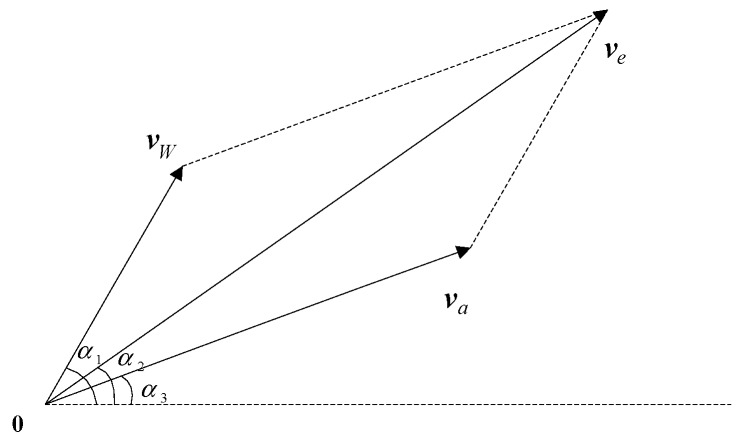
Wind speed synthetic relationship.

As shown in Figure 2, via the wind speed, the airship velocity relative to the Earth can be expressed as

(3) $$v_{e}=v_{a}+v_{W}$$

where $v_{e}$ and $v_{a}$ denote the airship velocities relative to the Earth and atmosphere, $\alpha_{1}$ is the angle between the horizontal axis and wind speed $v_{W}$, and $\alpha_{2}$ and $\alpha_{3}$ are the angles from $v_{e}$ and $v_{a}$ to the horizontal axis, respectively.

### 2.2. System State Equation of SINS/GNSS/ADS Integrated Navigation

The base coordinate system for establishment of the system state model is the E–N–U geography coordinate system. The system state X(t) of SINS/GNSS/ADS is defined as

(4) $$X\left(t\right)=\left[\begin{array}{ccc} x_{SINS}\left(t\right) & x_{BD}\left(t\right) & x_{ADS}\left(t\right) \end{array}\right]\;X\left(t\right)=\left[\begin{array}{ccc} x_{SINS}\left(t\right) & x_{BD}\left(t\right) & x_{ADS}\left(t\right) \end{array}\right]\;x_{SINS}\left(t\right)={\left[\phi_{E}\;\phi_{N}\;\phi_{U}\;\delta v_{SE}\;\delta v_{SN}\;\delta v_{SU}\;\delta L_{S}\;\delta \lambda_{S}\;\delta h_{S}\right]}^{T}\;x_{GNSS}\left(t\right)={\left[\delta v_{GE}\;\delta v_{GN}\;\delta v_{GU}\;\delta L_{G}\;\delta \lambda_{G}\;\delta h_{G}\right]}^{T}\;x_{ADS}\left(t\right)={\left[\delta v_{WcE}\;\delta v_{WcN}\;\delta v_{WcU}\;\delta v_{WrE}\;\delta v_{WrN}\;\delta v_{WrU}\right]}^{T}$$

where $\phi_{E}$, $\phi_{N}$ and $\phi_{U}$ represent the platform misalignment angle of SINS; $\delta v_{SE}$, $\delta v_{SN}$ and $\delta v_{SU}$ the velocity error of SINS; $\delta \lambda_{S}$, $\delta L_{S}$ and $\delta h_{S}$ the position error of SINS; $\delta v_{GE}$, $\delta v_{GN}$ and $\delta v_{GU}$ the velocity error of GNSS; $\delta \lambda_{G}$, $\delta L_{G}$ and $\delta h_{G}$ the position error of GNSS; $\delta v_{WcE}$, $\delta v_{WcN}$ and $\delta v_{WcU}$ the constant wind speed error; and $\delta v_{WrE}$, $\delta v_{WrN}$ and $\delta v_{WrU}$ the random wind speed error.

The system state equation is

(5) $$\dot{X}\left(t\right)=f\left(X\left(t\right)\right)+W\left(t\right)$$

where f(X(t)) is a nonlinear function of the state and is expressed by

(6) $$f\left(X\left(t\right)\right)=\left[\begin{array}{l} C_{\omega }^{-1}\left[\left(I-C_{n}^{c}\right){\hat{\omega }}_{in}^{n}+C_{n}^{c}\delta \omega_{in}^{n}-C_{b}^{c}\delta \omega_{ib}^{b}\right]\\ \left[I-{\left(C_{n}^{c}\right)}^{T}\right]C_{b}^{c}{\hat{f}}_{sf}^{b}+{\left(C_{n}^{c}\right)}^{T}C_{b}^{c}\delta f_{sf}^{b}-\left(2\delta \omega_{ie}^{n}+\delta \omega_{en}^{n}\right)\times v\\ \;-\left(2{\hat{\omega }}_{ie}^{n}+{\hat{\omega }}_{en}^{n}\right)\times \delta v+\left(2\omega_{ie}^{n}+\omega_{en}^{n}\right)\times \delta v+\delta g\\ \frac{v_{N}}{R_{M}+h}-\frac{\left(v_{N}-\delta v_{N}\right)}{\left(R_{M}-\delta R_{M}\right)+\left(h-\delta h\right)}\\ \frac{v_{E}\sec L}{R_{N}+h}-\frac{\left(v_{E}-\delta v_{E}\right)\sec\left(L-\delta L\right)}{\left(R_{N}-\delta R_{N}\right)+\left(h-\delta h\right)}\\ \delta v_{U}\\ -\delta v_{GE}/\tau_{GvE}\\ -\delta v_{GN}/\tau_{GvN}\\ -\delta v_{GU}/\tau_{GvU}\\ -\delta L_{G}/\tau_{GL}\\ -\delta \lambda_{G}/\tau_{G\lambda }\\ -\delta h_{G}/\tau_{Gh}\\ O_{3\times 1}\\ -\delta v_{WrE}/\tau_{wE}\\ -\delta v_{WrN}/\tau_{wN}\\ -\delta v_{WrU}/\tau_{wU} \end{array}\right]$$

where $C_{\omega }^{}$ is the Euler platform error angle matrix; $C_{n}^{c}$ and $C_{b}^{c}$ are the attitude transformation matrices; δg is the errors of gravity; $\delta \omega_{ib}^{b}$ is the measurement error of the gyro; $\omega_{ie}^{n}$ is the rotational angular velocity of the Earth; $\omega_{en}^{n}$ is the angular velocity of the vehicle relative to the Earth; $\omega_{ie}^{n}$ is the rotational angular velocity of the Earth; ${\hat{\omega }}_{ie}^{n}$, ${\hat{\omega }}_{en}^{n}$ and ${\hat{\omega }}_{in}^{n}$ are the actual values of $\omega_{ie}^{n}$, $\omega_{en}^{n}$ and $\omega_{in}^{n}$ in the actual navigation frame; $\delta \omega_{ie}^{n}$, $\delta \omega_{en}^{n}$ and $\delta \omega_{in}^{n}$ represent the corresponding errors; $\delta v_{i}$ represents the velocity error in the corresponding direction; L and h are the longitude and height of the airship; δL and δh are the errors of L and h, respectively; $\tau_{i}$ (i=E,N,U) is the relevant time; ${\hat{f}}_{sf}^{b}$ and $\delta f_{sf}^{b}$ are the specific force and its associated error, respectively; $R_{M}$ and $R_{N}$ are the meridian and prime vertical radiuses of curvature; and $\delta R_{M}$ and $\delta R_{N}$ are the errors of $R_{M}$ and $R_{N}$, respectively.

The system noise vector W(t) is described as

(7) $$W\left(t\right)={\left[W_{i}\right]}^{T}\;i=1,2,\cdots ,21$$

where $W_{i},\;i=1,2,\cdots ,21$ are the random noise of the state.

### 2.3. Measurement Equation of SINS/GNSS/ADS Integrated Navigation

SINS/GNSS/ADS integration consists of the SINS/GNSS subsystem and SINS/ADS subsystem. The measurement equation of the SINS/GNSS subsystem is obtained based on the position information integration, which is expressed by

(8) $$z_{1}\left(t\right)=\left[\begin{array}{c} \left(L_{S}-L_{G}\right)R_{M}+V_{\delta L}\\ \left(\lambda_{S}-\lambda_{G}\right)R_{N}\cos L+V_{\delta \lambda }\\ h_{S}-h_{G}+V_{\delta h} \end{array}\right]$$

where $L_{S}$, $\lambda_{S}$ and $h_{S}$ are the latitude, longitude and altitude of SINS, respectively; $L_{G}$, $\lambda_{G}$ and $h_{G}$ are the latitude, longitude and altitude of GNSS, respectively; and $V_{\delta L}$, $V_{\delta \lambda }$ and $V_{\delta h}$ are the errors of the GNSS. It should be noted that the errors of these sensors are considered to be known in this paper.

The measurement equation of SINS/ADS subsystem is expressed by

(9) $$v_{A}=\left[\begin{array}{ccc} 1 & \phi_{U} & -\phi_{N}\\ -\phi_{U} & 1 & \phi_{E}\\ \phi_{N} & -\phi_{E} & 1 \end{array}\right]\left[\begin{array}{c} v_{E}+v_{wcE}+v_{wrE}\\ v_{N}+v_{wcN}+v_{wrN}\\ v_{U}+v_{wcU}+v_{wrU} \end{array}\right]$$

(10) $$\begin{array}{l} v_{AE}=v_{E}+v_{wcE}+v_{wrE}+\phi_{U}v_{N}+\phi_{U}v_{wcN}+\phi_{U}v_{wrN}-\phi_{N}v_{U}-\phi_{N}v_{wcU}-\phi_{N}v_{wrU}\\ v_{AN}=-\phi_{U}v_{E}-\phi_{U}v_{wcE}-\phi_{U}v_{wrE}+v_{N}+v_{wcN}+v_{wrN}+\phi_{E}v_{U}+\phi_{E}v_{wcU}+\phi_{E}v_{wrU}\\ v_{AU}=\phi_{N}v_{E}+\phi_{N}v_{wcE}+\phi_{N}v_{wrE}-\phi_{E}v_{N}-\phi_{E}v_{wcN}-\phi_{E}v_{wrN}+v_{U}+v_{wcU}+v_{wrU} \end{array}$$

(11) $$\begin{array}{ll} z_{2}\left(t\right) & =\left[\begin{array}{l} v_{SE}-v_{ADSE}\\ v_{SN}-v_{ADSN}\\ v_{SU}-v_{ADSU} \end{array}\right]\\ & =\left[\begin{array}{l} \delta v_{E}-v_{wcE}-v_{wrE}-\phi_{U}v_{N}-\phi_{U}v_{wcN}-\phi_{U}v_{wrN}+\phi_{N}v_{U}+\phi_{N}v_{wcU}+\phi_{N}v_{wrU}+V_{vE}\\ \delta v_{N}+\phi_{U}v_{E}+\phi_{U}v_{wcE}+\phi_{U}v_{wrE}-v_{wcN}-v_{wrN}-\phi_{E}v_{U}-\phi_{E}v_{wcU}-\phi_{E}v_{wrU}+V_{vN}\\ \delta v_{U}-\phi_{N}v_{E}-\phi_{N}v_{wcE}-\phi_{N}v_{wrE}+\phi_{E}v_{N}+\phi_{E}v_{wcN}+\phi_{E}v_{wrN}-v_{wcU}-v_{wrU}+V_{vU} \end{array}\right] \end{array}$$

where $v_{EI}$, $v_{NI}$ and $v_{UI}$ are the velocities of SINS and $v_{EA}$, $v_{NA}$ and $v_{UA}$ are the velocities of ADS. It should be noted that the velocity used in this paper is relative to the geographic frame and is calculated from the airspeed.

The system measurement equation of SINS/GNSS/ADS integration is described as

(12) $$Z\left(t\right)=\left[\begin{array}{l} z_{1}\left(t\right)\\ z_{2}\left(t\right) \end{array}\right]=h\left(X\left(t\right)\right)+V\left(t\right)$$

where

(13) $$h\left(X\left(t\right)\right)=\left[\begin{array}{l} R_{M}\cdot \delta L\\ R_{N}\cos L\cdot \delta \lambda \\ \delta h\\ \delta v_{E}-v_{wcE}-v_{wrE}-\phi_{U}v_{N}-\phi_{U}v_{wcN}-\phi_{U}v_{wrN}+\phi_{N}v_{U}+\phi_{N}v_{wcU}+\phi_{N}v_{wrU}\\ \delta v_{N}+\phi_{U}v_{E}+\phi_{U}v_{wcE}+\phi_{U}v_{wrE}-v_{wcN}-v_{wrN}-\phi_{E}v_{U}-\phi_{E}v_{wcU}-\phi_{E}v_{wrU}\\ \delta v_{U}-\phi_{N}v_{E}-\phi_{N}v_{wcE}-\phi_{N}v_{wrE}+\phi_{E}v_{N}+\phi_{E}v_{wcN}+\phi_{E}v_{wrN}-v_{wcU}-v_{wrU} \end{array}\right]$$

(14) $$V\left(t\right)={\left[V_{\delta L}\;V_{\delta \lambda }\;V_{\delta h}\;V_{vE}\;V_{vN}\;V_{vU}\right]}^{T}$$

Thus, (5) and (12) provide the mathematical model for SINS/GNSS/ADS integrated navigation.

### 2.4. Wind Field-Based Constraint Model

In the most cases of airship flight, the vertical wind speed in the stratosphere is stable and close to a constant value (~20 km/s), and the airship does not change the altitude often. Thus, for simplicity, the vertical wind speed is neglected and it is also assumed that the altitude of the airship remains constant. Based on this, the airship state error equation can be written as [31]

(15) $$\left\{ \begin{array}{l} \delta \dot{x}=\delta v_{a}\cos\alpha_{3}+\delta v_{WE}=\delta v_{e}\cos\alpha_{2}\\ \delta \dot{y}=\delta v_{a}\sin\alpha_{3}+\delta v_{WN}=\delta v_{e}\sin\alpha_{2} \end{array}\right.$$

where $\delta \dot{x}$ and $\delta \dot{y}$ are the airship velocity error on the x and y axes, respectively; $\delta v_{a}$ and $\delta v_{e}$ are the airship velocity errors relative to the atmosphere and Earth; $\delta v_{WE}$ and $\delta v_{WN}$ are the projections of the wind speed on the East and North directions, respectively, and ω is the angle rate.

By combining (15) with (4), the constraint equation can be expressed as

(16) DX=d

where the state constraint matrix D and the constraint vector quantity d are expressed as

(17) $$D=\left[\begin{array}{ccccccccc} 0 & 0 & 0 & \cos\phi_{U} & 0 & 0 & 0 & 0 & 0\\ 0 & 0 & 0 & 0 & \sin\phi_{U} & 0 & 0 & 0 & 0 \end{array}\right]$$

(18) $$d={\left[\begin{array}{cc} \delta v_{WE}+\delta v_{a}\cos\phi_{U} & \delta v_{WN}+\delta v_{a}\sin\phi_{U} \end{array}\right]}^{T}$$

where $\delta v_{WE}$ and $\delta v_{WN}$ are the wind speeds in the East and North directions, respectively.

## 3. Constrained Unscented Particle Filter

### 3.1. Conventional Unscented Particle Filter

Consider the state equation of a discrete system

(19) $$X_{k}=f\left(X_{k-1}\right)+W_{k}$$

where $X_{k-1}$ denotes the state vector at epoch k−1, f(⋅) is a nonlinear function, and $W_{k}$ is the process noise.

The measurement equation of the discrete system is

(20) $$Z_{k}=h\left(X_{k}\right)+V_{k}$$

where $Z_{k}$ denotes the measurement vector, h(⋅) is also a nonlinear function, and $V_{k}$ is the measurement noise.

The conventional UPF includes the following steps.

**Step 1.** Initialization: k=0

For i=1,2,⋯,N, draw the states $X_{0}^{i}$ from the prior $p\left(X_{0}^{i}\right)$ and let

(21) $${\bar{X}}_{0}^{i}=E\left[X_{0}^{i}\right]$$

(22) $$P_{0}^{i}=E\left[(X_{0}^{i}-{\bar{X}}_{0}^{i}){\left(X_{0}^{i}-{\bar{X}}_{0}^{i}\right)}^{T}\right]$$

The sigma points can be selected as

(23) $$\left\{ \begin{array}{l} \omega_{0}^{\left(m\right)}\;=\;\frac{1}{n_{a}+\lambda }\\ \omega_{0}^{\left(c\right)}=\;\frac{1}{n_{a}+\lambda }+(1+\alpha^{2}+\beta )\\ \omega_{i}^{\left(m\right)}=\omega_{i}^{\left(c\right)}=\frac{1}{2(n_{a}+\lambda )}i=1,2,\ldots ,2N \end{array}\right.$$

where $\omega_{i}^{\left(m\right)}$ and $\omega_{i}^{\left(c\right)}$ are the importance weights of the mean and covariance; α is a coefficient to control the distribution of sampling points; β is a non-negative weighting coefficient for describing the prior distribution of **X**; λ is a scaling parameter; and $n_{a}=n_{X}+n_{W}+n_{V}$ is the dimension of the augmented state, where $n_{X}$, $n_{W}$ and $n_{V}$ are the dimensions of state vector $X_{k}$, process noise $W_{k}$ and measurement noise $V_{k}$, respectively.

**Step 2.** For k=1,2,⋯

(I) Importance sampling
  For i=1,2,⋯,N, update the particles with UKF:
  (a) Calculate the sigma points
    (24) $$\chi_{k-1}^{i}=\left[{\bar{X}}_{k-1}^{i}\;{\bar{X}}_{k-1}^{i}+\sqrt{\left(n_{a}+\lambda \right)P_{k-1}^{i}}\;{\bar{X}}_{k-1}^{i}-\sqrt{\left(n_{a}+\lambda \right)P_{k-1}^{i}}\right]$$
  (b) Time update
    (25) $$\chi_{k-1}^{i}=f\left(\chi_{k-1}^{iX},\chi_{k-1}^{iW}\right)$$
    (26) $$\chi_{k-1}^{i}=f\left(\chi_{k-1}^{iX},\chi_{k-1}^{iW}\right)$$
    (27) $$P_{k|k-1}^{i}=\sum_{j=0}^{2n_{a}}w_{j}^{\left(c\right)}\left[\chi_{j,k|k-1}^{iX}-{\bar{X}}_{k|k-1}^{i}\right]{\left[\chi_{j,k|k-1}^{iX}-{\bar{X}}_{k|k-1}^{i}\right]}^{T}$$
    (28) $$Z_{k|k-1}^{i}=h\left(\chi_{k|k-1}^{iX},\chi_{k-1}^{iV}\right)$$
    (29) $${\bar{Z}}_{k|k-1}^{i}=\sum_{j=0}^{2n_{a}}w_{j}^{\left(m\right)}Z_{j,k|k-1}^{i}$$
  (c) Measurement update
    (30) $$P_{Z}=\sum_{j=0}^{2n_{a}}w_{j}^{\left(c\right)}\left[Z_{j,k|k-1}^{i}-{\bar{Z}}_{k|k-1}^{i}\right]{\left[Z_{j,k|k-1}^{i}-{\bar{Z}}_{k|k-1}^{i}\right]}^{T}$$
    (31) $$P_{XZ}=\sum_{j=0}^{2n_{a}}w_{j}^{\left(c\right)}\left[\chi_{j,k|k-1}^{i}-{\bar{X}}_{k|k-1}^{i}\right]{\left[Z_{j,k|k-1}^{i}-{\bar{Z}}_{k|k-1}^{i}\right]}^{T}$$
    (32) $$K_{k}=P_{XZ}P_{Z}^{-1}$$
    (33) $${\bar{X}}_{k}^{i}={\bar{X}}_{k|k-1}^{i}+K_{k}\left(Z_{k}^{}-{\bar{Z}}_{k|k-1}^{i}\right)$$
    (34) $${\hat{P}}_{k}^{i}=P_{k|k-1}^{i}-K_{k}P_{Z}K_{k}^{T}$$
    The particles are sampled by ${\hat{X}}_{k}^{i}∼q\left(X_{k}^{i}|X_{0:k-1}^{i},Z_{1:k}^{}\right)=N\left({\bar{X}}_{k}^{i},{\hat{P}}_{k}^{i}\right)$. Subsequently, set ${\hat{X}}_{0:k-1}^{i}∼\left(X_{0:k-1}^{i},X_{k}^{i}\right)$ and ${\hat{P}}_{0:k-1}^{i}∼\left(P_{0:k-1}^{i},P_{k}^{i}\right)$, and normalize the importance weights.
    (35) $$\omega_{k}^{i}=\omega_{k-1}^{i}\frac{p\left(Z_{k}|{\hat{X}}_{k}^{i}\right)p\left({\hat{X}}_{k}^{i}|X_{k-1}^{i}\right)}{q\left({\hat{X}}_{k}^{i}|X_{0:k-1}^{i},Z_{1:k}\right)}$$
    (36) $${\tilde{\omega }}_{k}^{i}=\omega_{k}^{i}/\left(\sum_{j=0}^{N}\omega_{k}^{j}\right)$$
(II) Resampling
  Ignore the samples ${\hat{X}}_{0:k}^{i}$ with low importance weights. To obtain N random samples $X_{0:k}^{i}$ approximately distributed according to $p\left({\hat{X}}_{0:k}^{i}|Z_{1:k}^{}\right)$, we duplicate the particles having high weights and set ${\tilde{\omega }}_{k}^{i}=\omega_{k}^{i}=N^{-1}$.
(III) Output
  (37) $${\tilde{X}}_{k}^{i}=\sum_{i=1}^{N}\omega_{k}^{i}X_{k}^{i}$$
  (38) $$P_{k}^{i}=\sum_{i=1}^{N}\omega_{k}^{i}(X_{k}^{i}-{\tilde{X}}_{k}^{i}){\left(X_{k}^{i}-{\tilde{X}}_{k}^{i}\right)}^{T}$$

### 3.2. Convergence of Constrained UPF

Suppose system state X is subject to the following constraint

(39) DX=d

where D denotes the state constraint matrix and d the constraint vector quantity.

If the estimate ${\tilde{X}}_{k}^{i}$ of UPF is projected on the constraint surface, this minimum projection ${\tilde{X}}_{k}^{i}{}^{*}$ is given as (for clarity, we substitute ${\tilde{X}}_{k}^{i}$ and ${\tilde{X}}_{k}^{i}{}^{*}$ with $\tilde{x}$ and ${\tilde{x}}^{*}$, respectively)

(40) $${\tilde{x}}^{*}=\min{\left({\tilde{x}}^{*}-\tilde{x}\right)}^{T}\Sigma^{-1}\left({\tilde{x}}^{*}-\tilde{x}\right)$$

such that

(41) $$D{\tilde{x}}^{*}=d$$

To solve the above minimum problem, the Lagrange function is constructed as

(42) $$L={\left({\tilde{x}}^{*}-\tilde{x}\right)}^{T}\Sigma^{-1}\left({\tilde{x}}^{*}-\tilde{x}\right)+2\lambda^{T}\left(D{\tilde{x}}^{*}-d\right)$$

From (42), we get

(43) $$\Sigma^{-1}\left({\tilde{x}}^{*}-\tilde{x}\right)+D^{T}\lambda =0$$

and

(44) $$D{\tilde{x}}^{*}-d=0$$

According to (43), we obtain

(45) $$\begin{array}{l} \Sigma^{-1}\left({\tilde{x}}^{*}-\tilde{x}\right)+D^{T}\lambda =0\\ \Sigma^{-1}\left({\tilde{x}}^{*}-\tilde{x}\right)=-D^{T}\lambda \\ {\tilde{x}}^{*}-\tilde{x}=-\Sigma D^{T}\lambda \end{array}$$

From (45), we readily have

(46) $${\tilde{x}}^{*}=D^{-1}d$$

Substituting (46) into (45) yields

(47) $$\begin{array}{l} D^{-1}d-\tilde{x}=-\Sigma D^{T}\lambda \\ d-D\tilde{x}=-D\Sigma D^{T}\lambda \\ D\Sigma D^{T}\lambda =D\tilde{x}-d\\ \lambda ={\left(D\Sigma D^{T}\right)}^{-1}\left(D\tilde{x}-d\right) \end{array}$$

From (45) we also have

(48) $${\tilde{x}}^{*}=\tilde{x}-\Sigma D^{T}\lambda$$

Substituting (47) into (48) yields

(49) $${\tilde{x}}^{*}=\tilde{x}-\Sigma D^{T}{\left(D\Sigma D^{T}\right)}^{-1}\left(D\tilde{x}-d\right)$$

Thus, the state estimate by the constrained UPF can be obtained as

(50) $${\tilde{X}}_{k}^{i*}={\tilde{X}}_{k}^{i}-\Sigma D^{T}{\left(D\Sigma D^{T}\right)}^{-1}\left(D{\tilde{X}}_{k}^{i}-d\right)$$

where ${\tilde{X}}_{k}^{i}$ denotes the system state estimation from UPF.

From the above it can be seen that the state estimate given by (50) is actually an optimal solution under the constraint of the wind field model expressed by (16).

### 3.3. Convergence of Constrained UPF

**Lemma** **1.** *For the nonlinear dynamic system described by (19) and (20), the relationship among nonlinear function* f *, prior distribution* $p_{po}{}_{\left(k-1|k-1\right)}$ *, and posterior distribution* $p_{pr}{}_{\left(k|k-1\right)}$ *of state* X *can be expressed as*

(51) $$\begin{array}{ll} \left(p_{po}{}_{\left(k|k-1\right)},f\right) & =\int p_{po}{}_{\left(k-1|k-1\right)}p_{pr}{}_{\left(k|k-1\right)}fdx\\ & =\left(p_{po}{}_{\left(k-1|k-1\right)},p_{pr}{}_{\left(k|k-1\right)}f\right) \end{array}$$

The proof of Lemma 1 can be found in Appendix A.

**Lemma** **2.** *Assume that for any function* f *and constant* c *,*

(52) $$E\left[{\left(\left(p_{po}{}_{\left(k-1|k-1\right)}^{N},f\right)-\left(p_{po}{}_{\left(k-1|k-1\right)}^{},f\right)\right)}^{2}\right]\leq c\frac{{\left\|f\right\|}^{2}}{N}$$

*Then, we readily have*

(53) $$E\left[{\left(\left(p_{po}{}_{\left(k|k-1\right)}^{N},f\right)-\left(p_{po}{}_{\left(k|k-1\right)}^{},f\right)\right)}^{2}\right]\leq c\frac{{\left\|f\right\|}^{2}}{N}$$

The proof of Lemma 2 can be found in Appendix B.

**Lemma** **3.** *Given the system equation defined by (19), the relationship among nonlinear function* f *, prior distribution* $p_{po}{}_{\left(k|k-1\right)}$ *, and posterior distribution* $p_{po}{}_{\left(k|k\right)}$ *of state* X *can also be expressed as*

(54) $$\left(p_{po}{}_{\left(k|k\right)},f\right)=\frac{\left(p_{po}{}_{\left(k|k-1\right)},hf\right)}{\left(p_{po}{}_{\left(k|k-1\right)},h\right)}$$

The proof of Lemma 3 can be found in Appendix C.

**Theorem** **1.** *Given the system equation defined by (19), for all* k>0 *, there exists a constant* $c_{1}$ *, which is independent of* N *, such that for any* f *,*

(55) $$E\left[{\left(\left(p_{po\left(k|k\right)}^{N},f\right)-\left(p_{po}{}_{\left(k|k\right)}^{},f\right)\right)}^{2}\right]\leq c_{1}\frac{{\left\|f\right\|}^{2}}{N}$$

The proof of Theorem 1 can be found in Appendix D.

## 4. Simulations and Analysis

Simulations were conducted to evaluate the performance of the proposed constrained UPF algorithm for a SINS/GNSS/ADS integrated airship navigation system. Comparison analysis with the extended Kalman filter (EKF) and the robust adaptive UPF (RAUPF) [29,30] was also conducted to demonstrate the improved performance of the proposed algorithm.

Suppose the airship with a SINS/GNSS/ADS integrated navigation system is flying in the stratosphere. The airship moves to the East at a speed of 20 m/s, and the initial position of the airship is at East longitude 108.9°, North latitude 34.2°, and altitude 20 km. The flight time is 800 s. The Earth’s rotation angular velocity is $\omega_{ie}=15{\;}^{o}/h$, the radius of the earth is $R_{e}=6378\;\mathrm{km}$, and the acceleration of gravity is $g=9.780\;m/s^{2}$. The sampling periods of SINS, GNSS, and ADS are 0.01 s, 0.2 s, and 1 s, respectively. The filtering period is 1s. The initial position error is ($\delta \lambda =0^{\prime },\;\delta L=0^{\prime },\;\delta h=0\;m$) and the velocity error is $\delta v_{E}=\delta v_{N}=\delta v_{U}=0.1$ m/s. The gyro’s random drift and walk are 0.1°/h and 0.01°/$\sqrt{h}$, respectively. The accelerometer’s zero offset and random walk are $10^{-3}g$ and $5\times 10^{-4}g/\sqrt{s}$, respectively. The horizontal and altitude positioning errors of GNSS are 0.6 m and 2 m, the barometric altimeter error is 20 m, and the velocity error is 2 m/s. The airspace above sea level within the altitude of 20 km is mainly dominated by west wind with a speed below 20 m/s. The gust wind in the airspace is in the North by East 10° with the speed of 0–2 m/s.

To study the performance of the proposed constrained UPF, trials were conducted under different average wind speeds of 10 m/s, 15 m/s, and 20 m/s, respectively. The initial state variance, system noise covariance and observation noise covariance are set as

(56) $$\begin{array}{l} P_{0}=diag\{{\left(0.1^{\prime }\right)}^{2},\;{\left(0.1^{\prime }\right)}^{2},\;{\left(0.1^{\prime }\right)}^{2},\;{\left(0.1m/s\right)}^{2},\;{\left(0.1m/s\right)}^{2},\;{\left(0.1m/s\right)}^{2},\;{\left(0m\right)}^{2},\;{\left(0m\right)}^{2},\;{\left(0m\right)}^{2},\\ \;{\left(2m/s\right)}^{2},\;{\left(2m/s\right)}^{2},\;{\left(2m/s\right)}^{2},\;{\left(0.6m\right)}^{2},\;{\left(0.6m\right)}^{2},\;{\left(2m\right)}^{2},\\ \;{\left(9.8m/s\right)}^{2},\;{\left(1.7m/s\right)}^{2},\;{\left(0.1m/s\right)}^{2},\;{\left(1.9m/s\right)}^{2},\;{\left(0.3m/s\right)}^{2},\;{\left(0.1m/s\right)}^{2}\}\\ P_{0}=diag\{{\left(0.1^{\prime }\right)}^{2},\;{\left(0.1^{\prime }\right)}^{2},\;{\left(0.1^{\prime }\right)}^{2},\;{\left(0.1m/s\right)}^{2},\;{\left(0.1m/s\right)}^{2},\;{\left(0.1m/s\right)}^{2},\;{\left(0m\right)}^{2},\;{\left(0m\right)}^{2},\;{\left(0m\right)}^{2},\\ \;{\left(2m/s\right)}^{2},\;{\left(2m/s\right)}^{2},\;{\left(2m/s\right)}^{2},\;{\left(0.6m\right)}^{2},\;{\left(0.6m\right)}^{2},\;{\left(2m\right)}^{2},\\ \;{\left(9.8m/s\right)}^{2},\;{\left(1.7m/s\right)}^{2},\;{\left(0.1m/s\right)}^{2},\;{\left(1.9m/s\right)}^{2},\;{\left(0.3m/s\right)}^{2},\;{\left(0.1m/s\right)}^{2}\}\\ R_{1}=diag\left\{{\left(0.6m\right)}^{2},\;{\left(0.6m\right)}^{2},\;{\left(2m\right)}^{2},\;{\left(9.8m/s\right)}^{2},\;{\left(1.7m/s\right)}^{2}\;{\left(0.1m/s\right)}^{2}\right\}\\ R_{2}=diag\left\{{\left(0.6m\right)}^{2},\;{\left(0.6m\right)}^{2},\;{\left(2m\right)}^{2},\;{\left(14.8m/s\right)}^{2},\;{\left(2.6m/s\right)}^{2}\;{\left(0.1m/s\right)}^{2}\right\}\\ R_{3}=diag\left\{{\left(0.6m\right)}^{2},\;{\left(0.6m\right)}^{2},\;{\left(2m\right)}^{2},\;{\left(19.7m/s\right)}^{2},\;{\left(3.5m/s\right)}^{2}\;{\left(0.1m/s\right)}^{2}\right\} \end{array}$$

where $R_{i}(i=1,2,3)$ stands for the observation noise covariances for the cases of different average wind speeds.

**Figure 3:**
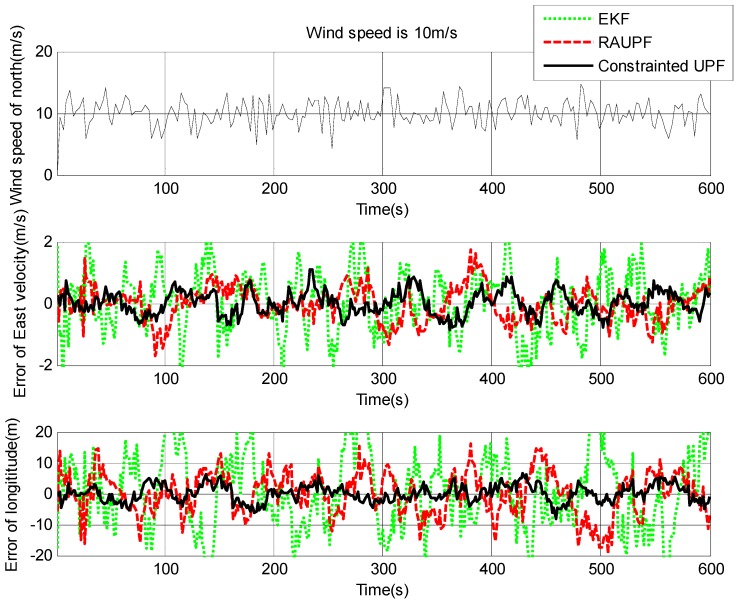
East velocity and longitude errors under the wind speed of 10 m/s.

**Figure 4:**
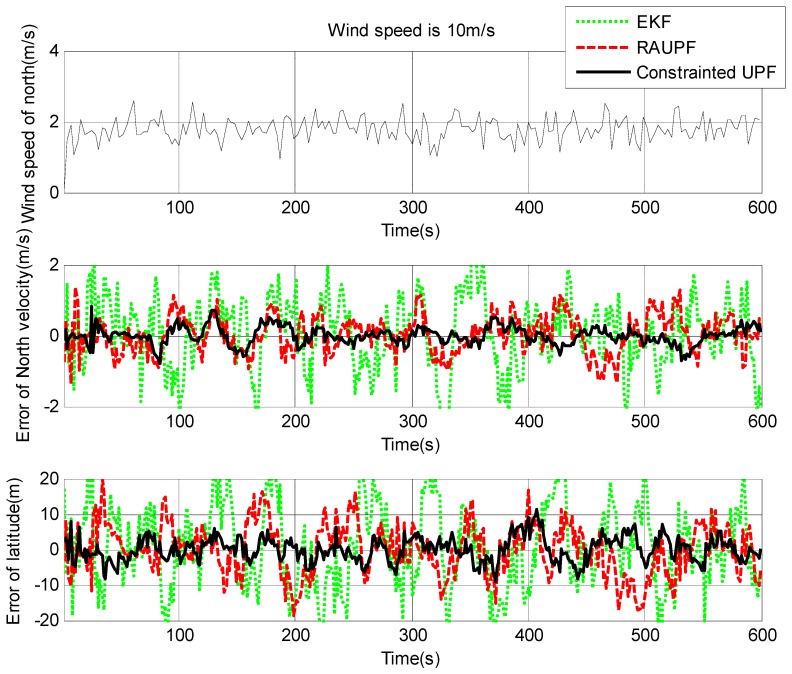
North velocity and latitude errors under the wind speed of 10 m/s.

**Figure 5:**
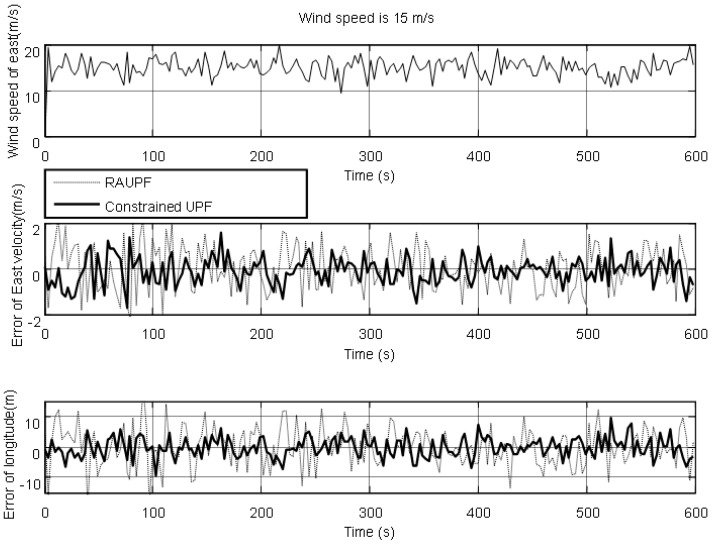
East velocity and longitude errors under the wind speed of 15 m/s.

**Figure 6:**
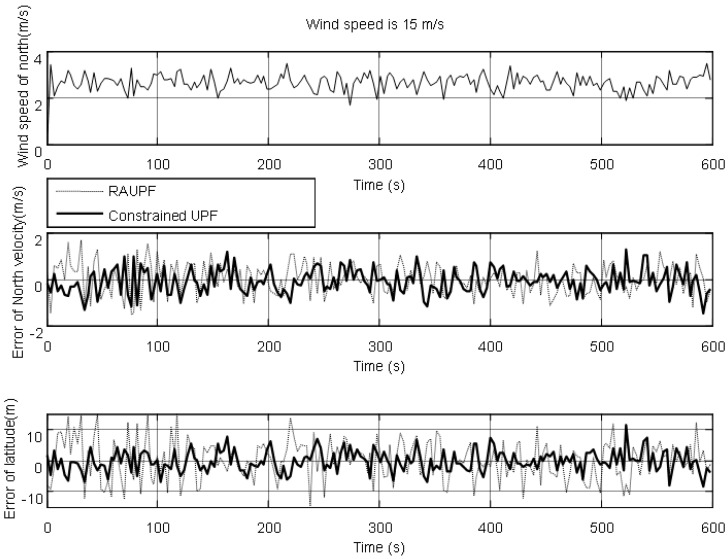
North velocity and latitude errors under the wind speed of 15 m/s.

**Figure 7:**
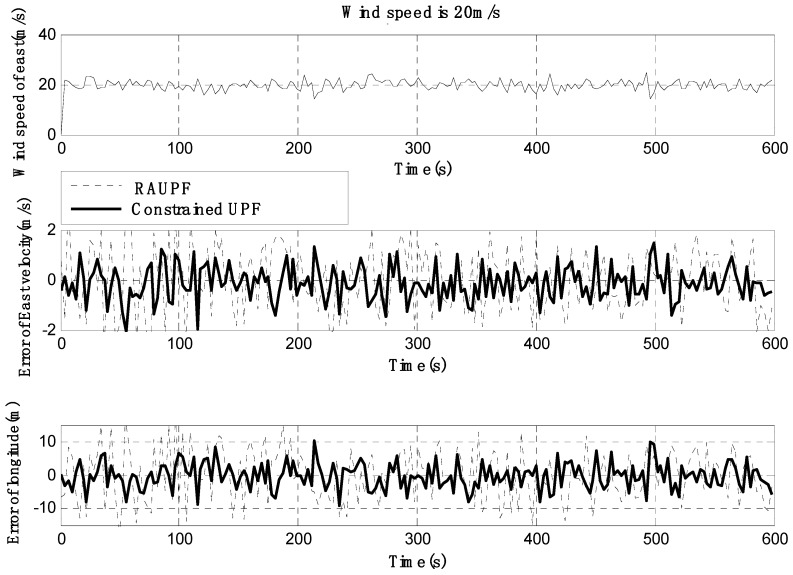
East velocity and longitude errors under the wind speed of 20 m/s.

**Figure 8:**
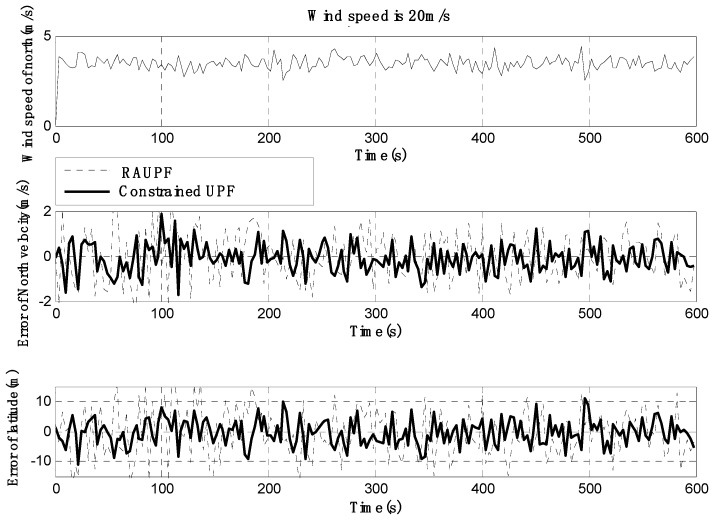
North velocity and latitude errors under the wind speed of 20 m/s.

The estimated velocity and position errors under different wind speeds are shown in Figure 3, Figure 4, Figure 5, Figure 6, Figure 7 and Figure 8, and their corresponding mean values are listed in Table 1, Table 2 and Table 3. In the case of 10 m/s constant wind, during the time period from 0 s to 600 s, the velocity and position errors obtained by EKF beyond ±2 m/s and ±20 m, respectively. This is because the linearization of system model causes a large navigation error. RAUPF improves the performance of EKF. The velocity and position errors obtained by RAUPF are within (−0.7 m/s, +0.7 m/s) and (−7 m, +7 m). They are also much larger than those errors by the constrained UPF, which are within (−0.3 m/s, +0.3 m/s) and (−4 m, +4 m). Considering that the performance of EKF is too poor for the wind field disturbance, in the following we shall focus on the comparison of RAUPF with the proposed constrained UPF for the cases of 15 m/s and 20 m/s wind speeds. The similar trend can also be observed for these two cases. In the case of 15m/s constant wind, the velocity and position errors obtained by RAUPF are within (−1.1 m/s, +1.1 m/s) and (−8 m, +8 m), while those by the constrained UPF are within (−0.6 m/s, +0.6 m/s) and (−5.5 m, +5.5 m). In the case of 20 m/s constant wind, the velocity and position errors obtained by RAUPF are within (−1.6m/s, +1.6 m/s) and (−9.5 m, +9.5 m), while those by the constrained UPF are within (−0.7 m/s, +0.7 m/s) and (−6 m, +6 m). Therefore, it is evident that the velocity and position errors obtained by the proposed constrained UPF are much smaller than those by RAUPF.

It is also observed that the latitude and North velocity errors are smaller than the longitude and East velocity for RAUPF. The reason is that the gust wind is in the North by East 10° and thus its East velocity component is larger than its North velocity component, more greatly affecting the East velocity and longitude of the airship. However, the constrained UPF does not suffer from such an effect caused by the wind field disturbance. The North velocity and latitude are in the similar accuracy as the East velocity and longitude, without any obvious disturbance by the wind field. This demonstrates that the proposed constrained UPF is able to resist the disturbance of wind field.

In addition, only a slight change in the accuracy of the constrained UPF was observed due to the increase of wind speed. This means that the ability of the constrained UPF to suppress wind field disturbance becomes stronger with the increase of the wind speed.

**Figure 9:**
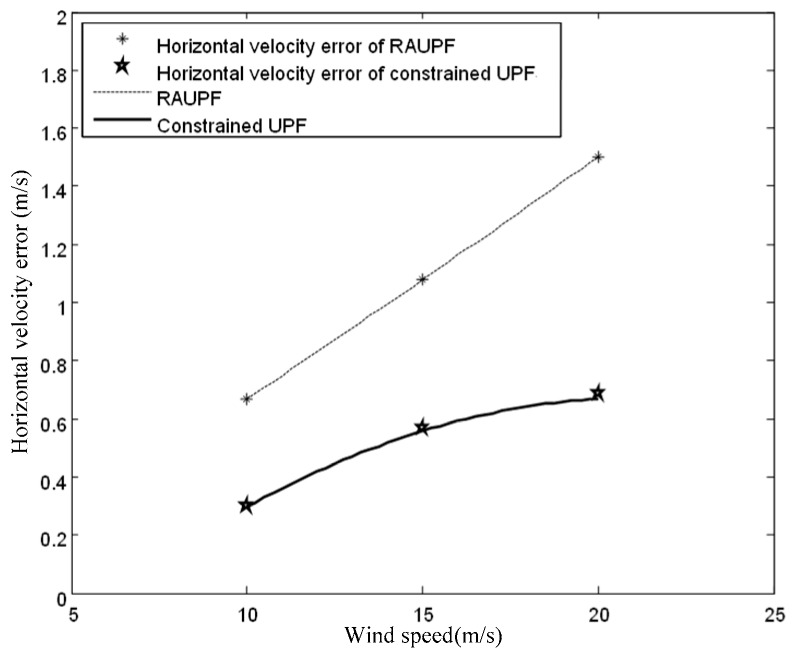
Horizontal velocity errors for both RAUPF and constrained UPF.

**Figure 10:**
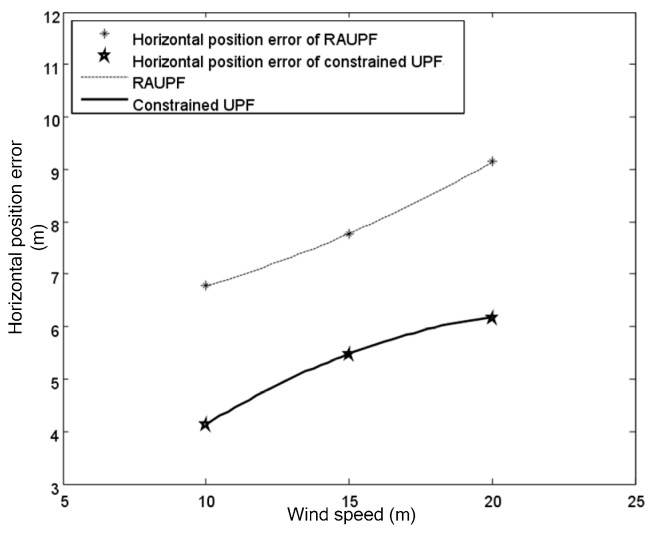
Horizontal position errors for both RAUPF and constrained UPF.

Figure 9 and Figure 10 show the fitting curves for the horizontal velocity and position errors of the airship by both RAUPF and constrained UPF. It can be seen that the horizontal velocity and position errors obtained by RAUPF are linearly increased with the increase of the wind speed. This demonstrates that RAUPF lacks the capability to resist the wind disturbance. In contrast, the slopes of the fitting curves of the horizontal velocity and position errors by the constrained UPF are decreased, demonstrating that the larger the wind speed is the stronger UPF’s resistance to the wind disturbance.

The above simulation results demonstrate that the proposed constrained UPF can effectively inhibit the disturbance of wind field, leading to improved positioning accuracy for SINS/GNSS/ADS integrated airship navigation in comparison with RAUPF.

## 5. Conclusions

This paper presents a new constrained UPF for SINS/GNSS/ADS integration to improve the performance of airship positioning and navigation under the disturbance of wind field. The contributions of this paper are that (i) the wind speed model and navigation mathematical model are established for SINS/GNSS/ADS integration; and (ii) a constrained UPF is developed using the wind speed model as a constraint to fuse SINS and GNSS measurements to generate system state estimation for airship navigation based on SINS/GNSS/ADS integration, leading to the optimal state estimation in the presence of wind disturbance. Simulations and comparison analysis verify that the proposed constrained UPF can effectively inhibit the influence of wind field, leading to the improved accuracy comparing to EKF and ARUPF for SINS/GNSS/ADS integrated airship navigation in the presence of wind disturbance.

Future research work will focus on two aspects. One is the experimental evaluation of the proposed constrained UPF. Practical experiments on airship flight based on SINS/GNSS/ADS integrated navigation will be conducted to further evaluate the performance of the proposed algorithm. The other is on improvement of the proposed constrained UPF. The proposed algorithm will be combined with advanced artificial intelligence technologies such as pattern recognition, neural network, and advanced expert systems, thus establishing an intelligent algorithm to automatically deal with the disturbances of wind field for the airship navigation.

## Figures and Tables

**Table 1:** Mean errors of velocity and position under the wind speed of 10 m/s.

Filtering Methods	East Velocity Error (m/s)	North Velocity Error (m/s)	Longitude Error (m)	Latitude Error (m)
EKF	0.8532	0.7955	8.2235	8.3465
RAUPF	0.5679	0.3324	4.6657	4.7968
Constrained UPF	0.2123	0.2198	2.8123	2.9456

**Table 2:** Mean errors of velocity and position under the wind speed of 15 m/s.

Filtering Methods	East Velocity Error (m/s)	North Velocity Error (m/s)	Longitude Error (m)	Latitude Error (m)
RAUPF	0.8136	0.6180	5.4120	5.5852
Constrained UPF	0.3058	0.4767	3.8606	3.8769

**Table 3:** Mean errors of velocity and position under the wind speed of 20 m/s.

Filtering Methods	East Velocity Error (m/s)	North Velocity Error (m/s)	Longitude Error (m)	Latitude Error (m)
RAUPF	1.1127	1.0092	6.8033	6.4456
Constrained UPF	0.5269	0.4388	4.5319	4.1869

## Appendix A. Proof of Lemma 1

**Proof.** The prior distribution and the posterior distribution of state X are

(A1) $$p_{pr}=p\left(X\right)$$

(A2) $$p_{po}=p\left(X|Z\right)$$

According to the prediction of Bayes recursions, we have

(A3) $$p_{po}{}_{\left(k|k-1\right)}=\int p_{po}{}_{\left(k-1|k-1\right)}p_{pr}{}_{\left(k|k-1\right)}dx$$

It can be easily seen from (59) that for any function f,

(A4) $$\begin{array}{ll} \left(p_{po}{}_{\left(k|k-1\right)},f\right) & =\int p_{po}{}_{\left(k-1|k-1\right)}p_{pr}{}_{\left(k|k-1\right)}fdx\\ & =\left(p_{po}{}_{\left(k-1|k-1\right)},p_{pr}{}_{\left(k|k-1\right)}f\right) \end{array}$$

## Appendix B. Proof of Lemma 2

**Proof.** According to Lemma 1, we have

(A5) $$\begin{array}{l} \;\left|\left(p_{po}{}_{\left(k|k-1\right)}^{N},f\right)-\left(p_{po}{}_{\left(k|k-1\right)}^{},f\right)\right|\\ =\left|\left(p_{po}{}_{\left(k-1|k-1\right)}^{N},p_{pr\left(k|k-1\right)}f\right)-\left(p_{po}{}_{\left(k-1|k-1\right)}^{},p_{pr\left(k|k-1\right)}f\right)\right| \end{array}$$

Then,

(A6) $$\begin{array}{l} \;E\left[{\left(\left(p_{po}{}_{\left(k|k-1\right)}^{N},f\right)-\left(p_{po}{}_{\left(k|k-1\right)}^{},f\right)\right)}^{2}\right]\\ =E\left|\left(p_{po}{}_{\left(k-1|k-1\right)}^{N},p_{pr\left(k|k-1\right)}f\right)-\left(p_{po}{}_{\left(k-1|k-1\right)}^{},p_{pr\left(k|k-1\right)}f\right)\right|\\ \leq c\frac{{\left\|p_{pr\left(k|k-1\right)}f\right\|}^{2}}{N} \end{array}$$

Since $\left\|p_{pr\left(k|k-1\right)}f\right\|\leq \left\|f\right\|$, we can obtain

(A7) $$\begin{array}{l} \;E\left[{\left(\left(p_{po}{}_{\left(k|k-1\right)}^{N},f\right)-\left(p_{po}{}_{\left(k|k-1\right)}^{},f\right)\right)}^{2}\right]\\ \leq c\frac{{\left\|p_{pr\left(k|k-1\right)}f\right\|}^{2}}{N}\\ \leq c\frac{{\left\|f\right\|}^{2}}{N} \end{array}$$

## Appendix C. Proof of Lemma 3

**Proof.** According to the updating process of Bayes recursion, we have

(A8) $$p_{po}{}_{\left(k|k\right)}=\frac{p_{po}{}_{\left(k|k-1\right)}h}{\int p_{po}{}_{\left(k|k-1\right)}h\;dx}$$

It is easily to see from (62) that for any function f,

(A9) $$\left(p_{po}{}_{\left(k|k\right)},f\right)=\frac{\int p_{po}{}_{\left(k|k-1\right)}hf\;dx}{\int p_{po}{}_{\left(k|k-1\right)}h\;dx}=\frac{\left(p_{po}{}_{\left(k|k-1\right)},hf\right)}{\left(p_{po}{}_{\left(k|k-1\right)},h\right)}$$

## Appendix D. Proof of Theorem 1

**Proof.** By Lemmas 1–3, we have

(A10) $$\begin{array}{l} \;\left(p_{po\left(k|k\right)}^{N},f\right)-\left(p_{po}{}_{\left(k|k\right)}^{},f\right)\\ =\frac{\left(p_{po\left(k|k-1\right)}^{N},hf\right)}{\left(p_{po\left(k|k-1\right)}^{N},h\right)}-\frac{\left(p_{po}{}_{\left(k|k-1\right)}^{},hf\right)}{\left(p_{po}{}_{\left(k|k-1\right)}^{},h\right)}\\ =\frac{\left(p_{po\left(k|k-1\right)}^{N},hf\right)}{\left(p_{po\left(k|k-1\right)}^{N},h\right)}-\frac{\left(p_{po\left(k|k-1\right)}^{N},hf\right)}{\left(p_{po\left(k|k-1\right)}^{},h\right)}+\frac{\left(p_{po\left(k|k-1\right)}^{N},hf\right)}{\left(p_{po\left(k|k-1\right)}^{},h\right)}-\frac{\left(p_{po}{}_{\left(k|k-1\right)}^{},hf\right)}{\left(p_{po}{}_{\left(k|k-1\right)}^{},h\right)} \end{array}$$

where,

(A11) $$\begin{array}{l} \;\left|\frac{\left(p_{po\left(k|k-1\right)}^{N},hf\right)}{\left(p_{po\left(k|k-1\right)}^{N},h\right)}-\frac{\left(p_{po\left(k|k-1\right)}^{N},hf\right)}{\left(p_{po\left(k|k-1\right)}^{},h\right)}\right|\\ =\frac{\left(p_{po\left(k|k-1\right)}^{N},hf\right)}{\left(p_{po\left(k|k-1\right)}^{},h\right)\left(p_{po\left(k|k-1\right)}^{N},h\right)}\left|\left(p_{po\left(k|k-1\right)}^{},h\right)-\left(p_{po\left(k|k-1\right)}^{N},h\right)\right|\\ \leq \frac{\left\|f\right\|}{\left(p_{po\left(k|k-1\right)}^{},h\right)}\left|\left(p_{po\left(k|k-1\right)}^{},h\right)-\left(p_{po\left(k|k-1\right)}^{N},h\right)\right| \end{array}$$

Using Minkowski’s inequality, we can obtain

(A12) $$\begin{array}{l} \;E{\left[{\left(\left(p_{po\left(k|k\right)}^{N},f\right)-\left(p_{po}{}_{\left(k|k\right)}^{},f\right)\right)}^{2}\right]}^{\frac{1}{2}}\\ \leq E{\left[{\left(\frac{\left(p_{po\left(k|k-1\right)}^{N},hf\right)}{\left(p_{po\left(k|k-1\right)}^{N},h\right)}-\frac{\left(p_{po\left(k|k-1\right)}^{N},hf\right)}{\left(p_{po\left(k|k-1\right)}^{},h\right)}\right)}^{2}\right]}^{\frac{1}{2}}\\ \;+E{\left[{\left(\frac{\left(p_{po\left(k|k-1\right)}^{N},hf\right)}{\left(p_{po\left(k|k-1\right)}^{},h\right)}-\frac{\left(p_{po}{}_{\left(k|k-1\right)}^{},hf\right)}{\left(p_{po}{}_{\left(k|k-1\right)}^{},h\right)}\right)}^{2}\right]}^{\frac{1}{2}}\\ \leq \frac{\left\|f\right\|}{\left(p_{po\left(k|k-1\right)}^{},h\right)}E{\left[{\left(\left(p_{po\left(k|k\right)}^{N},h\right)-\left(p_{po}{}_{\left(k|k\right)}^{},h\right)\right)}^{2}\right]}^{\frac{1}{2}}\\ \;+\frac{1}{\left(p_{po\left(k|k-1\right)}^{},h\right)}E{\left[{\left(\left(p_{po\left(k|k-1\right)}^{N},hf\right)-\left(p_{po}{}_{\left(k|k-1\right)}^{},hf\right)\right)}^{2}\right]}^{\frac{1}{2}}\\ \leq \frac{\left\|f\right\|}{\left(p_{po\left(k|k-1\right)}^{},h\right)}\sqrt{c}\frac{\left\|h\right\|}{\sqrt{N}}+\frac{\left\|h\right\|}{\left(p_{po\left(k|k-1\right)}^{},h\right)}\sqrt{c}\frac{\left\|f\right\|}{\sqrt{N}}\\ \leq \frac{2\sqrt{c}\left\|h\right\|}{\left(p_{po\left(k|k-1\right)}^{},h\right)}\frac{\left\|f\right\|}{\sqrt{N}} \end{array}$$

Letting $\sqrt{c_{1}}=\frac{2\sqrt{c}\left\|h\right\|}{\left(p_{po\left(k|k-1\right)}^{},h\right)}$ yields

(A13) $$E\left[{\left(\left(p_{po\left(k|k\right)}^{N},f\right)-\left(p_{po}{}_{\left(k|k\right)}^{},f\right)\right)}^{2}\right]\leq c_{1}\frac{{\left\|f\right\|}^{2}}{N}$$

Thus, if there exists a probability density $p_{po}{}_{\left(k-1|k-1\right)}^{N}$ to approximate posterior density $p_{po}{}_{\left(k-1|k-1\right)}^{}$, we can find proposal distribution $p_{po\left(k|k\right)}^{N}$ to approximate posterior density $p_{po}{}_{\left(k|k\right)}^{}$.
